# Characteristics of intracardiac electrogram of the interventricular septum in the left bundle branch pacing

**DOI:** 10.1186/s12872-022-02715-5

**Published:** 2022-06-17

**Authors:** Xiaojie Cai, Longfu Jiang, Shanshan Zhuo, Hao Wu

**Affiliations:** Department of Cardiology, HwaMei Hospital, University of Chinese Academy of Sciences, Ningbo, China

**Keywords:** Case report, Left bundle branch pacing, Intrinsic, Intracardiac electrogram, Interventricular septum

## Abstract

**Background:**

Left bundle branch pacing (LBBP) has become a hot topic in the field of physiological pacing. However, only a few studies have described the characteristics of the intrinsic intracardiac electrogram (EGM) while placing the left bundle branch (LBB) lead.

**Case presentation:**

Herein, we reported a case with atrial premature contractions to the ventricle during the LBBP procedure. Paced and intrinsic (supraventricular) EGMs were recorded and analyzed.

**Conclusions:**

The myocardium of the interventricular septum could be divided into four regions based on electrophysiology: the right septal area, the left septal area, the endocardium of the left ventricular septum, and the LBB area. This might guide the electrophysiological localization of the LBB lead in the septum.

## Background

Huang et al. [1] successfully described the first case, left bundle branch pacing (LBBP) in 2017, and since then, it has become a hot topic in the field of physiological pacing. Several studies have investigated that the intracardiac electrogram (EGM) of LBBP is characterized by native left bundle branch (LBB) potential (Po_LBB_) and distinct isoelectric stimulus-QRS interval with pacing [2, 3]. However, the importance of paced QRS morphology in electrocardiogram (ECG) to demonstrate the capture of LBB is controversial with respect to the heterogenicity in the complexes [4, 5], and the characteristics of the intrinsic intracardiac EGM representing the myocardial electrical activity at the location of the lead tip during the implantation of the LBB lead are rarely described. Herein, we reported a case of intermittent atrial premature contractions that captures the ventricle when the pacing LBB lead is screwed. The paced and intrinsic (supraventricular) surface and intracardiac EGMs were recorded simultaneously. The data explained the phenomenon of trans-septal paced and native electrophysiology and guided the positioning of the LBB lead in the interventricular septum (IVS).

## Case presentation

A 60-year-old male patient was admitted for recurrent dizziness with chest discomfort for 1 year. He had no history of other chronic diseases, medication, or surgeries. The coronary CTA showed mild atherosclerosis in the coronary artery. Transthoracic echocardiography indicated an enlarged left atrium, and his left ventricular ejection fraction was 67%. Dynamic ECG showed sinus bradycardia with an average heart rate of 45 beats per min, second degree type II sinoatrial block, and the longest time of sinus arrest as 3.1 s. ECG did not indicate intraventricular conduction delay. Thus, the transvenous pacemaker implantation with LBBP was scheduled. The study was approved by HwaMei Hospital, University of Chinese Academy of Science, Ningbo, China (YJ-KYSB-NBEY-2021-079-01).

**Fig. 1:**
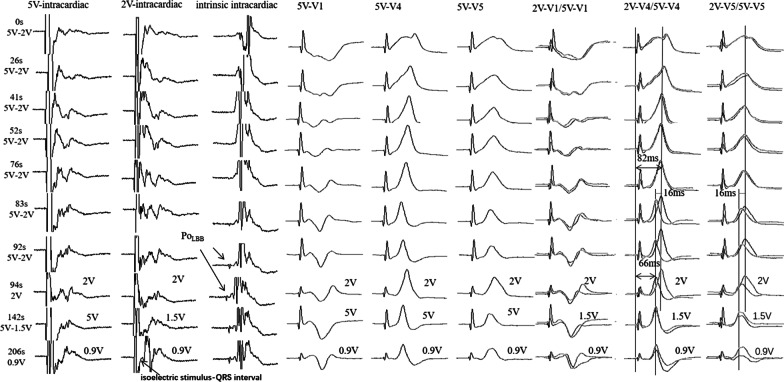
Morphology of paced and intrinsic (supraventricular) EGM during LBB lead implantation. 0–76 s: constant pLVAT in leads V4/V5 at 5 V/2 V@0.5 ms output. 83 s: pLVAT was jumped and shortened from 82 to 66 ms at 5 V@0.5 ms output, and indistinct Po_LBB_ appeared. 94 s: pLVAT was jumped and shortened from 82 to 66 ms at 2 V@0.5 ms output, and Po_LBB_ became clear. 142 S: constant pLVAT at the high and low outputs (5 V/1.5 V@0.5 ms, 5 V/0.9 V@0.5 ms). 206 s: recorded isoelectric stimulus-QRS interval. EGM, electrogram; LBB, left bundle branch; pLVAT, stimulus-peak left ventricular activation time; Po_LBB_, LBB potential

A dual-chamber pacemaker (model A3DR01; Medtronic Inc., Minneapolis, MN, USA) was implanted in the patient. The LBBP lead (model 3830–69 cm; Medtronic Inc.) was delivered using a sheath (C315, Medtronic Inc.) via the left subclavian vein access. About 206 s were spent screwing the LBBP lead in place successfully. During the trans-septal process of the LBB lead, continuous unipolar pacing at 2 V@0.5 ms was performed to observe the paced QRS morphology of the surface ECG. The impedance was monitored dynamically. A 16-ms shortening jump of stimulus-peak left ventricular activation time (pLVAT) from 82 to 66 ms in lead V5 at 5 V@0.5 ms output and indistinct Po_LBB_ were observed at 83 s. Typically, a high output (10 V) is used to measure pLVAT. However, the default output of the Medtronic device programmer for measuring the unipolar pacing impedance was 5 V, and during the measurement, ECG was recorded to observe whether pLVAT was shortened abruptly compared with 2 V@0.5 ms. Therefore, we used 5 V as a high output. As the lead continued to be screwed in for 94 s, the same jump of pLVAT at 2 V@0.5 ms output was recorded which remained constant at low output, and Po_LBB_ was distinct. The isoelectric stimulus-QRS interval was visible at 206 s (Fig. 1). Thus, it was determined that the tip of the lead reached the selective LBB location [6]. Unipolar pacing demonstrated that the final LBB threshold was 0.3 V@0.5 ms, left ventricular septal myocardial threshold was 0.5 V@0.5 ms, the R wave amplitude was 13 mV, and the unipolar impedance was 872 Ω.

**Fig. 2:**
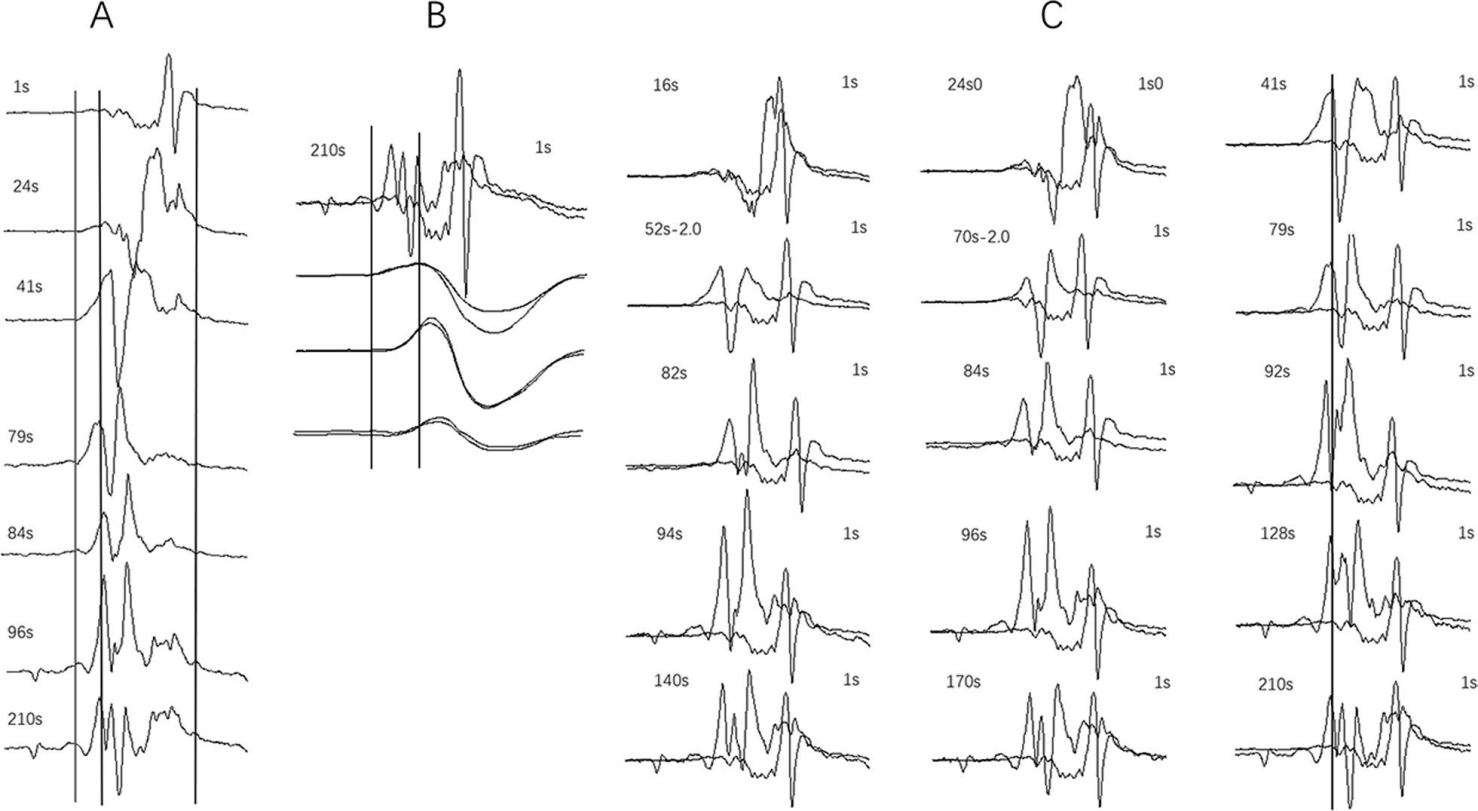
Morphology of intrinsic intracardiac EGM of the LBB lead tip during the LBBP procedure and comparison with the morphology before screwing in. **A** Morphology of intrinsic intracardiac EGM when the LBB lead tip was located at different sites in the interventricular septum. **B** Taking the mapping recorded at 210 s as an example to demonstrate the method of overlapping and comparing the morphologies of two different sites. The intrinsic QRS complexes on surface ECG recorded at 210 s and 1 s (before screwing into the interventricular septum) were matched, such that the morphologies of intracardiac EGMs at 210 s and 1 s were overlapped and compared to avoid time errors. **C** Comparison of the morphology of the intrinsic intracardiac EGM at different sites in the interventricular septum and before screwing (1 s) (marked as 2.0, which means the amplitude was halved). EGM, electrogram; LBB, left bundle branch; LBBP, left bundle branch pacing; ECG, electrocardiogram

Notably, the screwing procedure of the LBB lead always connected John-Jiang’s connecting cable. Unlike traditional cable that had to be detached to provide the rapid lead rotations, the Jiang cable could be revolved to record the EGM uninterrupted. As the LBB lead was placed transseptally from the right ventricular septal surface to the left, the tip of the lead was continuously paced to observe the changes in the paced QRS complexes. Thus, the intrinsic EGMs could not be recorded with sinus rhythm. This patient had some premature atrial beats capturing the ventricle (3–4:1) during the operation. Therefore, paced mixed intrinsic ventricular complexes from supraventricular impulses could be recorded. According to the comparison of the morphology, beat by beat, recorded from the right ventricular septum (RVS) to the endocardial side of the left ventricular septum (LVS) along the path of the LBB lead implantation, the intrinsic QRS did not show any obvious change on the surface ECG but had a significant difference on the intrinsic intracardiac EGM. Compared to the lead tip EGM on the right ventricular septal surface before the lead screwed in (1 s), the intrinsic intracardiac EGMs recorded before 41 s were dominated by a single posterior peak that moved forward gradually as the lead was screwed in continually. The waveform evolved into double peaks between 41 s and 94 s and changed from positive and negative to mainly positive. Subsequently, fragmentation potentials appeared at the rear. Then, the waveform emerged as several spikes until 210 s. After 84 s, the time from onset to the first peak remained unchanged (Fig. 2).

## Discussion and conclusion

LBBP is more effective than the right ventricular septal pacing in achieving synchronized activation of ventricles. The feasibility and safety of LBBP have been confirmed via short‐term and medium‐term follow‐ups in small cohorts [7]. A review showed that the incidence of AV block in patients with sick sinus syndrome was 8.4% during a mean follow-up period of 34.2 months [8]; hence, we decided to perform LBBP in this patient. The paced and intrinsic (supraventricular) surface and intracardiac EGMs were obtained simultaneously during the screwing of the lead from RVS to LVS. According to the characteristics of these patterns, we summarized the electrical distribution characteristics from RVS to LVS to guide the electrophysiological localization of the LBB lead in IVS.

Since the position of the supraventricular activation point was fixed and the trans-septal process had no effect on the conduction of supraventricular activation, we could avoid the error of overlapping comparison of intracardiac EGM morphology by overlapping the unchanged QRS complex of the ECGs at 1 s and other time points. According to the characteristics of intrinsic intracardiac EGMs combined with paced EGMs of the corresponding period, we speculated that the interventricular septal myocardium from the right side to the left side could be divided into four regions: the right septal area, the left septal area, the endocardium of LVS, and LBB area. The single posterior peak of the intrinsic intracardiac EGM recorded by the lead tip at 1 s represented the electrical activation of the right ventricle. Before 41 s, the tip of the lead was screwed to the left in the right septal area, and the peak representing the depolarization of the right ventricle moved forward gradually. This phenomenon confirmed the theory that the left ventricle depolarizes before the right ventricle [9]. Based on the appearance of the R wave in lead V1 at 41 s to the shortening jump of the pLVAT in lead V5 at 84 s and 5 V@0.5 ms output, the morphology of the right bundle branch block (RBBB) became perfect. The tip of the LBB lead was advanced in the left ventricular septal area, and the double peaks of the intrinsic intracardiac EGM represented the electrical activation of the left and right ventricles with the front position of the left ventricle. At 92 s, pLVAT shortened and remained constant at high and low outputs, indicating that the tip of the paced lead reached the endocardium of LVS and captured the left bundle branch and the adjacent local myocardium. The morphology of the intrinsic intracardiac EGM changed from positive and negative to mainly positive, and substantial fragmentation potentials appeared. Thus, we speculated that the amplitude of the potential in the endocardium of LVS was different from that in the right septal area. The depolarization of the myocardium in the right septal area was small, and when the lead tip passed through this area (1–43 s), the local electrical activation was submerged in the comprehensive ventricular excitation manifesting as a waveform with a single peak, and hence could not be displayed. The isoelectric stimulus-QRS interval appeared at 206 s, and the pacing selectively captured the LBB to excite the left ventricular myocardium. Next, we determined that the lead tip reached the LBB, and the threshold measured at this time was the LBB threshold [2]. Consecutively, the comprehensive myocardial excitation caused by the conduction system and the local myocardial excitation were distinct, which changed the pattern to increased spikes. In addition, we found that the peak time of left ventricular depolarization was not altered with the depth of the lead screwing in the IVS. The characteristics of the native electrical activity of IVS summarized above might aid in determining the direction and depth of the LBB lead screwed in during the procedure. The selective capture of the LBB is characterized by LBB potential, LBB current of injury in the unipolar EGM, constant pLVAT at high and low outputs, and isoelectric stimulus-QRS interval [10]. However, in this case, we observed that the isoelectric stimulus-QRS interval appeared later than the LBB potential, indicating that after the LBB potential is recorded during the implantation of the lead, it is possible to slowly screw the lead in a little for the isoelectric stimulus-QRS interval to capture LBB selectively.

In conclusion, according to the characteristics of intrinsic intracardiac EGM of IVS during the implantation of the LBB lead, the myocardium of IVS could be divided into four regions: the right septal area, the left septal area, the endocardium of LVS, and the LBB area. This might guide the electrophysiological localization of the LBB lead in the septum. However, this phenomenon needs to be substantiated with additional cases, and the electrophysiological mechanism needs to be explored further.

## Data Availability

All data generated or analyzed during this study are included in this published article [and its supplementary information files].

## Abbreviations

LBBP: Left bundle branch pacing
LBB: Left bundle branch
EGM: Electrogram
IVS: Interventricular septum
LVS: Left ventricular septum
RVS: Right ventricular septum
ECG: Electrocardiogram
CTA: Computed tomographic angiography
PoLBB: Left bundle branch potential
pLVAT: Stimulus-peak left ventricular activation time
RBBB: Right bundle branch block
